# SALOS—A UWB Single-Anchor Indoor Localization System Based on a Statistical Multipath Propagation Model

**DOI:** 10.3390/s24082428

**Published:** 2024-04-10

**Authors:** Sven Ole Schmidt, Marco Cimdins, Fabian John, Horst Hellbrück

**Affiliations:** Department of Electrical Engineering and Computer Science, Technische Hochschule Lübeck—University of Applied Sciences, Mönkhofer Weg 239, 23562 Lübeck, Germany; marco.cimdins@th-luebeck.de (M.C.); fabian.john@th-luebeck.de (F.J.); horst.hellbrueck@th-luebeck.de (H.H.)

**Keywords:** single-anchor localization, indoor localization, UWB, channel impulse response, multipath propagation model, optimal anchor positioning, signal processing, DW1000

## Abstract

Among other methods, UWB-based multi-anchor localization systems have been established for industrial indoor localization systems. However, multi-anchor systems have high costs and installation effort. By exploiting the multipath propagation of the UWB signal, the infrastructure and thus the costs of conventional systems can be reduced. Our UWB Single-Anchor Localization System (SALOS) successfully pursues this approach. The idea is to create a localization system with sophisticated signal modeling. Therefore, measured reference, like fingerprinting or training, is not required for position estimation. Although SALOS has already been implemented and tested successfully in an outdoor scenario with multipath propagation, it has not yet been evaluated in an indoor environment with challenging and hardly predictable multipath propagation. For this purpose, we have developed new algorithms for the existing hardware, mainly a three-dimensional statistical multipath propagation model for arbitrary spatial geometries. The signal propagation between the anchor and predefined candidate points for the tag position is modeled in path length and complex-valued receive amplitudes. For position estimation, these modeled signals are combined to multiple sets and compared to UWB measurements via a similarity metric. Finally, a majority decision of multiple position estimates is performed. For evaluation, we implement our localization system in a modular fashion and install the system in a building. For a fixed grid of 20 positions, the localization is evaluated in terms of position accuracy. The system results in correct position estimations for more than 73% of the measurements.

## 1. Introduction

Indoor localization is an ongoing research field with a plurality of solutions starting from light-based approaches [1], acoustics [2], or camera-based solutions [3] and ending with radio-based approaches. One key factor of success is performance and costs. Radio-based approaches with satisfactory performance often require costly infrastructure [4]. One approach in recent years is the reduction in costs by introducing single-anchor localization systems.

Single-anchor localization is a technique that aims to estimate the position of a target node in a wireless network using one single reference node, called the anchor [5]. This technique reduces the costs and complexity of conventional multi-anchor systems, which require multiple reference nodes and synchronization among them [4]. However, single-anchor localization also faces many challenges, such as the limited information available from one anchor, the multipath propagation of the wireless signals, and the non-line-of-sight conditions. To overcome these challenges, different approaches have been proposed, such as moving mobile anchors [6,7], integration of external inertial measurement units (IMUs) [8], applying advanced signal processing [9] and optimization methods [10,11], as well as exploiting multipath signals [12,13].

Despite the diverse approaches, the use of single-anchor localization systems has not yet become established. This may be because as hardware is minimized, the inaccuracy of position estimation increases. A deep understanding of the transmission conditions promotes localization quality and enables competitiveness with hardware-heavy established systems.

In this paper, we present our re-imagined novel system, SALOS, which is an ultra-wideband (UWB) single-anchor indoor localization system based on a statistical multipath propagation model. The idea is to create a localization system with sophisticated signal modeling. The resulting system works with a single anchor node. The localization of a tag with unknown position results from the comparison of UWB measurements with artificial modeled UWB receive signals at previously defined candidate points in the area. These receive signals are generated beforehand using only the floor plan of the room and the resulting multipath propagation. Therefore, neither measured reference, like fingerprinting, nor training is required for position estimation. The system is initialized via software purely. To the best of our knowledge, no such single-anchor localization system has been proposed yet.

**Figure 1:**
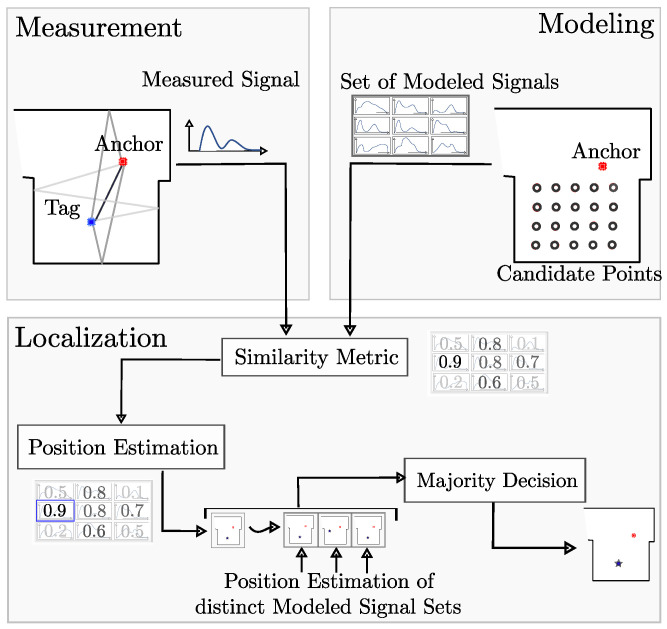
General structure of our single-anchor localization system SALOS: Modeled receive signals are compared with measurements to estimate the tag’s position accurately.

In [14], we have already shown with a rudimentary structure for the system that our approach to outdoor localization works with little multipath processing. For indoor use with massive multipath processing, we had to fundamentally re-develop the system. Figure 1 shows the structure of the system with the single processing steps from top to bottom. The main part is the modeling of sets of expected receive signals for possible tag positions, namely the candidate points (CPs), only based on the transmitted signal shape and the expected environment. Modeling is particularly challenging because the integrated low-cost hardware, namely Qorvo’s DW1000 RF chip, only provides, on the hardware side, post-processed receive signals, which are not physically correct, Qorvo Inc., Greensboro, NC, USA. The set of these modeled receive signals is compared with the measured signal by a similarity metric to estimate the corresponding most likely position of the tag. The most likely positions for multiple sets of modeled signals are evaluated via majority decision. This decision results in an overall candidate point, which will be the final estimated position for the measurement. Following this setup, no further external information during measurement is needed, such as a priori position estimations, IMU measurements, or multiple antennas. The only external information source is the floor plan of the localization environment.

The contributions of the paper are:

- We introduce our redesigned novel UWB single-anchor localization system, called SALOS, which works in massive multipath environments.
- For this, we present a three-dimensional multipath propagation model for arbitrary spatial geometries to construct receive signals with statistic variation in amplitude and phase.
- With these distinct modeled signals, we employ a straightforward algorithm and similarity metric to estimate the tag’s position.
- We evaluate the position accuracy of localization for a real indoor environment and publish the measurements and modeled datasets for free download.

The paper is organized as follows. Section 2 provides a review of related work on UWB-based localization systems with minimal infrastructure. A three-dimensional multipath propagation model is derived in Section 3. We explore UWB signal propagation in a multipath environment, resulting in modeled signals with statistical variation in amplitude and phase. Section 4 describes localization in detail, including anchor placement, signal processing of the measured signal with distance measurement, and position estimation. The evaluation setup, the metrics, and results for assessing localization performance are available in Section 5. The paper concludes with a summary and a discussion of future work with potential research directions.

## 2. Related Work

With SALOS—our novel UWB-based single-anchor localization system—we consider approaches for localization systems with a minimal infrastructure, and especially single-anchor systems, as related. Starting with Bluetooth, which has low costs, we will proceed with UWB systems and finally compare related work to our approach, focusing on single-anchor systems.

The radio technology Bluetooth offers localization capabilities with minimal infrastructure, as many Bluetooth radio chips are already built-in in many electronic devices such as smartphones. In [8], Ye et al. analyze angle estimation along with signal strength for distance estimation and IMU data for indoor localization. The authors present the feasibility of single-anchor localization with an accuracy of less than 1 m using Bluetooth. However, their approach requires customized hardware and IMU data. Since version 5.1, the Bluetooth standard offers direction finding via an optional constant tone extension, enabling low-cost localization for devices such as smartphones [15,16]. Both works demonstrate the versatility of single-anchor localization using Bluetooth. However, multipath propagation in indoor environments remains a challenge for narrowband radio technologies such as Bluetooth [16].

To address multipath propagation or even apply it for localization, we need higher bandwidth and short pulses. UWB communication systems meet these requirements. While SALOS works with active tag UWB nodes, the multipath-assisted MA-RTI system from [17] follows a passive approach. The system tracks objects with a localization accuracy of 1–2 m by incorporating multipath propagation, without nodes on the objects for localization. The approach is a good example how multipath propagation can help to improve accuracy or reduce costs. Still, this passive approach requires multiple anchors, and the localization accuracy may not be sufficient for localization of nodes on an object.

In [18], Ge et al. propose a single-anchor localization system that combines time difference-of-arrival (TDoA) from multiple antennas to calculate the phase difference-of-arrival (PDoA) for indoor localization. Wang et al. propose a model that also estimates the distance via TDoA, and the angle-of-arrival (AoA) for every tag’s candidate position [19]. Both works demonstrate that the single-anchor localization system achieves a localization accuracy within the decimeter level; however, both systems require customized hardware with multiple antennas for PDoA or AoA calculations. In this paper, we utilize only commercially off-the-shelf (COTS) available UWB radio chips, with a single built-in omnidirectional antenna for localization. In [20,21], Rzymowski et al. and Groth et al. proposed utilizing an electronically steerable parasitic array radiator (ESPAR) antenna for single-anchor localization. They show the feasibility of this single-anchor approach; however, the usage of an ESPAR antenna increases the complexity, and is not COTS available in many radio chips, such as Qorvo’s DW1000. Grosswindhager et al. present single-anchor localization with COTS available UWB radios [22]. In order to better evaluate the multipath propagation, the receive signal is artificially split up by using several antennas. Wang et al. propose a single-anchor localization with UWB signals, using time-of-flight and angle-of-arrival approaches in [23]. Although a single anchor is deployed, it is equipped with a circular antenna array, including multiple of Qorvo’s DW1000 RF chips. Different from our approach, all these systems try to handle multipath propagation by high-bandwidth signals and estimate the most likely direction of the tag and distance to the tag via the receive signal strength and multiple or special antennas.

In [24], Meissner et al. were the first to propose a two-dimensional single-anchor localization system and evaluate it via simulations. The idea is to exploit signal reflection on walls to calculate matching virtual anchors, which replace the requirement for physical anchors. We are inspired by this approach and use the virtual anchor concept in our modeling. Different to [24], we also evaluate our proposed system with measurements instead of simulations.

The aforementioned works rely upon multiple radios, additional measured external information, such as IMUs, or multiple or special antennas. To the best of our knowledge, there are only few works that do not depend on those restrictions. In [25], Mohammadmoradi et al. also implement their system with Qorvo’s DW1000 RF chip. They extract statistical features from the stored receive signal samples for usage in fingerprinting algorithms that require intensive training from a training measurement set. With this training-intensive approach, Mohammadmoradi et al. achieve localization accuracy in the decimeter range. The differences to our proposed approach are as follows. Instead of extracting statistical features from the whole receive signal, we only extract and utilize information from specific multipath components, potentially reducing computational complexity. Furthermore, our approach does not require a measured training dataset; it solely relies on floor map information and simulations.

In the following, we briefly summarize the difference of our approach. In this paper, we propose a novel approach that only employs measured information extracted from the DW1000’s UWB receive signal, previously modeled reference data, and no prior training. Therefore, our approach does not require special or multiple antennas; it works with the default antennas. In addition, we present a modular framework for the algorithmic implementation, enabling distributed computation on different machines and commissioning as a live system. Insights from our proposed signal processing and modeling of the multipath propagation of SALOS can be adapted to other approaches (e.g., radio technologies) that depend on multiple antennas in the future.

## 3. Construction of Receive Signals with a Three-Dimensional Multipath Model

With SALOS, we introduce a localization system that does not require any recorded measurement data like fingerprints as a reference. In our approach, the reference data for position estimation are modeled artificially. The multipath propagation of the transmit signal affects the UWB receive signals, which are the basis for our measurements. We explain the propagation of the signal to the receiver in Section 3.1. We introduce the correlation between the tag and anchor position and the receive signal in Section 3.2, which is the basis for our position estimation. We present details of our multipath model in Section 3.3, including the geometric model, the creation of complex-valued amplitude multi-path components, and the complete modeling of the receive signals.

### 3.1. UWB Signal Propagation in Multipath Environments

The position estimation accuracy of the localization system depends on the artificial reference data. To model realistic UWB receive signals as a reference, we require a deep understanding of signal propagation in environments. Therefore, we discuss the signal propagation from a UWB signal from an anchor to a tag and the resulting receive signal.

UWB is characterized by a spectrum with a minimum bandwidth of B≈500 MHz at a center frequency $f_{c}>3.1$ GHz [26]. We communicate in UWB low-band at $f_{c}\;=\;3.4944$ GHz with B=499.2 MHz, namely the IEEE 802.15.4-2011 standard channel 1. This band is provided by Qorvo’s DW1000 RF chip [27].

The transmit signal in the time domain is a sinc pulse multiplied with the carrier signal, so x(t) is defined as a cosine wave of frequency $f_{c}$, which is weighted with a sinc-function with zeros in the range of $T_{\mathrm{sinc}}=1/B$ around t=0:

(1) $$\begin{array}{c} x\left(t\right)=\frac{\sin(\pi \cdot t/T_{\mathrm{sinc}})}{\pi \cdot t/T_{\mathrm{sinc}}}\cdot \cos\left(2\pi \cdot f_{c}\cdot t\right), \end{array}$$

**Figure 2:**
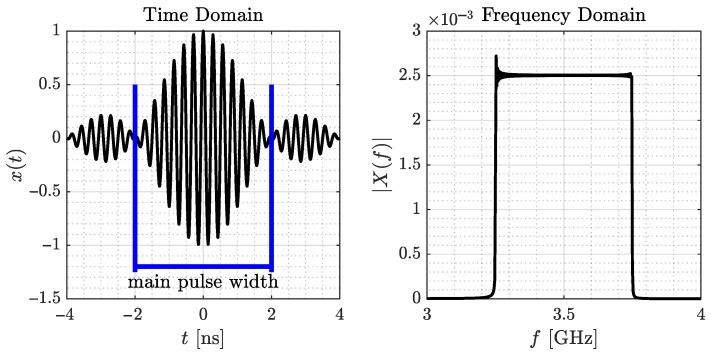
Transmit signal of UWB channel 1 in time and frequency domains.

Figure 2 plots x(t) in the time and frequency domains as X(f).

**Figure 3:**
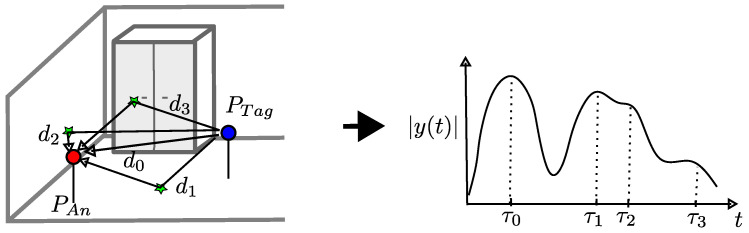
Left: Three-dimensional multipath environment including two walls, the ground, and furniture. Right: Resulting superposition of the signal reflections in receive signal y(t).

In the following, we assume an omnidirectional radiation pattern of the transmitting and receiving antennas. We selected this pattern because similar antenna patterns are installed in the actual implementation of the localization system. The receiver Rx receives x(t) multiple times following both the direct path and reflections on walls or obstacles. Figure 3 sketches this scene on the left-hand side. Here, $d_{0}$ is the direct path between Tx and Rx, $d_{1}$ is the path of reflection from the floor, $d_{2}$ is the path of reflection from the left wall, and $d_{3}$ is the path of reflection from the cabinet.

In general, each propagation results in a number *I* of reflected signals resulting from *I* paths. Each of the paths is characterized by several parameters: $\tau_{i}$ the transmission delay of the reflected signal at Rx, ${\underline{a}}_{i}$ the complex amplitude of the reflected signal, and $n_{i}$ the number of reflections while passing the corresponding *i*-th path. Delay $\tau_{i}=d_{i}/c_{0}$ is the fraction of the path length $d_{i}$ and the speed of light $c_{0}\approx 3\times 10^{8}\;\frac{m}{s}$. The amplitude ${\underline{a}}_{i}$ results from transmit power, reduced by propagation losses during transmission. Also, with each of the $n_{i}$ reflections, material and angle-dependent reflection losses occur due to the signal’s wall penetration.

Additionally, the reflected signal experiences a phase shift of $180^{\circ }$, and thus, the sign of its amplitude changes per reflection [28]. The channel impulse response (CIR) h(t) depicts the multipath’s influence on the transmission:

(2) $$\begin{array}{c} h\left(t\right)=\sum_{i=0}^{I-1}{\left(-1\right)}^{n_{i}}{\underline{a}}_{i}\cdot \delta \left(t-\tau_{i}\right) \end{array}$$

The signal y(t) at Rx is the superposition of all *I* reflected signals with:

(3)y(t)=x(t)∗h(t)(4)=∑i=0I−1(−1)nia_i·sin(π·(t−τi)/Tsinc)π·(t−τi)/Tsinc·cos(2π·fc·(t−τi)).

Equation (3) represents the receive signal in a multipath environment. The right-hand side of Figure 3 depicts a sketch of the superposed signal reflections in y(t) resulting from the multipath propagation, shown on the left.

We derive the correlation between the tag and anchor position and the receive signal in the next section.

### 3.2. Correlation between Anchor and Tag Positions and UWB Signals

To enable optimal analysis of the receive signal for tag position estimation, it is necessary to consider the correlation between the tag position $P_{\mathrm{Tag}}$ and signal y(t). In general, the paths of the received reflected signals depend on three factors: the anchor position $P_{\mathrm{An}}$, the spatial geometry, and the $P_{\mathrm{Tag}}$. For SALOS, the anchor is at a fixed position $P_{\mathrm{An}}$ within the spatial geometry, which is also assumed to be static, meaning the anchor position and spatial geometry remain unchanged and known.

**Figure 4:**
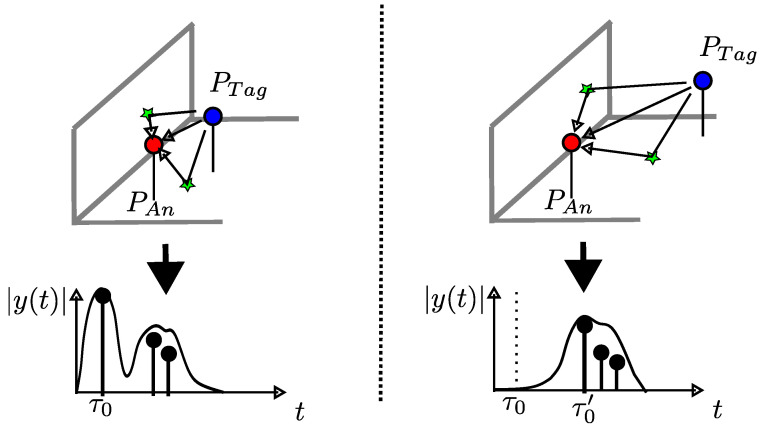
Influence of the tag position $P_{\mathrm{Tag}}$ on the receive signal.

Thus, changes in the receive signal and its origin’s multipath propagation result solely from changes of the position of the tag $P_{\mathrm{Tag}}$, as shown in Figure 4. This means that y(t) is correlated or mapped to $P_{\mathrm{Tag}}$. To reverse the correlation and reconstruct $P_{\mathrm{Tag}}$ from y(t), the mapping needs to be bijective. In this case, the y(t) of each considered $P_{\mathrm{Tag}}$ is unambiguous and is mapped to exactly one $P_{\mathrm{Tag}}$. If ambiguity occurs and the y(t) for multiple tag positions cannot be distinguished, additional measured external information is needed.

To ensure clear localization, we aim for bijective mapping for a specific set of CPs in which the tag is placed later on. In order to fulfill this condition, for initialization, the optimal anchor position $P_{\mathrm{An}}$ is selected in such a way that the receive signals are unambiguous at all CPs; see [13]. We discuss the positioning of the anchor in Section 4.1. The following section describes the modeling of receive signals.

### 3.3. Modeling of Receive Signals Based on a Given Spatial Geometry

For constructing sets of suitable artificial reference data, a realistic modeling of receive signals is needed. To accurately model receive signals for arbitrarily placed sensor nodes in a spatial geometry, following Equation (3), we need the original transmit signal x(t) and the modeled CIR $\hat{h}\left(t\right)$, including estimated transmission delays $\left\{{\hat{\tau }}_{i}\right\}$ and amplitudes $\left\{{\hat{\underline{a}}}_{i}\right\}$ for all reflected signals. First, we introduce a three-dimensional geometric model to calculate all reflected signals, including individual $\left\{{\hat{\tau }}_{i}\right\}$ in a multipath scenario, in Section 3.3.1. Then, we extend our model with estimates for the complex-valued amplitudes $\left\{{\hat{\underline{a}}}_{i}\right\}$ in Section 3.3.2. In the end, we show how the modeled receive signal is formed, see Section 3.3.3.

#### 3.3.1. Three-Dimensional Multipath Model for Transmission Delay Estimation

**Figure 5:**
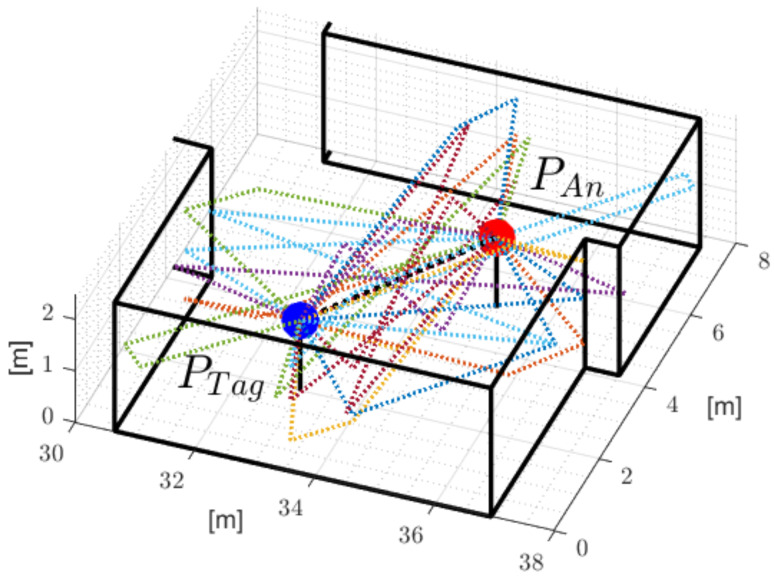
Estimated reflected signal paths of the proposed three-dimensional multipath propagation model.

To estimate the reflected signal’s transmission delays, we designed a three-dimensional multipath propagation model for arbitrary spatial geometries. Figure 5 depicts the estimated reflected signal paths for a given set of tag and anchor nodes on a floor.

**General Overview:** The three-dimensional spatial geometry contains multiple reflective surfaces (e.g., walls, ground, ceiling, furniture, and obstacles). We model the multipath propagation for each combination of anchor and tag position inside the geometry. With it, we calculate the respective path lengths and the reflection at the surfaces. Therefore, we assumed that the materials of the surfaces have no noticeable influence on the transmission delay of the reflected signals.

**Figure 6:**
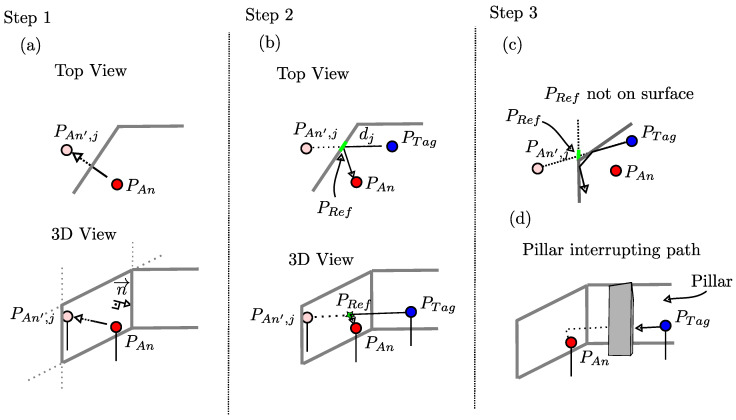
Three-dimensional multipath modeling of reflected signals with single reflection paths: (**a**) Step 1: Determination of the virtual anchors. (**b**) Step 2: Calculation of reflected signal’s path. (**c**) Step 3: Check for invalid paths. (**d**) Step 3: Check for interrupted paths.

Note that $R_{\max}$ will indicate the maximum number of reflections of all single signal paths. The following description outlines the modeling for paths with exact $R_{\max}\;=\;1$ reflection, illustrated in Figure 6, before the model is expanded to reflected signals with multiple reflections at the end of this section. **Note:** In the following, we distinguish between the infinite *plane* and the finite *surface* inside the plane.

**Step 1. Determining the position of virtual anchors:** For modeling the reflected signal paths, we determine the position of so-called virtual anchors, a reference point in the spatial geometry, to calculate the reflection path length between the anchor and the tag, and the reflection. For the *j*-th surface (j=1,...,J), the virtual anchor is the mirroring of the anchor on that surface. The mirroring of the anchor at position $P_{\mathrm{An}}$ on a plane is to be computed in two iterations, as shown in Figure 6a. First, determine the origin of the normal of the plane passing through the anchor position $P_{\mathrm{An}}$. Then determine the route (Δx, Δy, Δz) between the origin of the normal and the anchor itself. The virtual anchor of the *j*-th surface is on the other side of this plane, located at position $P_{{\mathrm{An}}^{\prime },j}=P_{\mathrm{An}}-2\cdot \left(\Delta x,\Delta y,\Delta z\right)$. Overall, for a spatial geometry with *J* walls, *J* virtual anchors result.

**Note:** For the calculation of a valid virtual anchor, it is not necessary that the origin of the normal be inside the surface.

**Step 2. Calculation of the reflected signal’s path:** To model the multipath propagation delay accurately, a single parameter per path is needed, namely the path length $d_{j}$ for the generation of h(t). We calculate the point $P_{\mathrm{Ref}}$ where the reflection occurs for the validity check of the path. These steps are sketched in Figure 6b.

Due to geometry, the path length $d_{j}$ is identical to the distance between the virtual anchor and the tag:

(5) $$\begin{array}{c} d_{j}=d_{\ell 2}\left(P_{\mathrm{Tag}}-P_{{\mathrm{An}}^{\prime },j}\right), \end{array}$$

where $d_{\ell 2}\left(\cdot \right)$ represents the Euclidean distance. For the point $P_{\mathrm{Ref}}$ where the reflection occurs, we draw a line between the virtual anchor and the tag. The intersection between the plane or the surface and the line is equal to $P_{\mathrm{Ref}}$. The resulting path leads from $P_{\mathrm{Tag}}$ over $P_{\mathrm{Ref}}$ to $P_{\mathrm{An}}$.

**Step 3. Check path for validity:** Not all paths created in the way described above are valid and thus distort the modeled receive signal. The first case occurs if the reflection $P_{\mathrm{Ref}}$ happens in the plane but outside the surface. Figure 6c sketches the case where the $P_{\mathrm{Ref}}$ is on the plane but not on the surface, resulting in an invalid path.

The second case is when a given surface (an obstacle, e.g., a pillar) is interrupting the reflected signal path, as shown in Figure 6d. With a single reflection, the path divides into two straight lines: one from $P_{\mathrm{An}}$ to $P_{\mathrm{Ref}}$ and the second from $P_{\mathrm{Ref}}$ to $P_{\mathrm{Tag}}$. For both lines, we check if one of the remaining surfaces k≠j intersects with any line. First, we check if there is an intersection with any surface’s plane. If this is true, the respective intersection is tested for whether it is inside the plane’s surface. If this is the case for at least one line and one surface, the path becomes invalid.

**Expansion of the modeling for $R_{\max}\;⩾\;2$:**

The exclusive integration of paths with a single reflection does not adequately represent reality. Also, paths with multiple reflections need to be taken into account. To model such paths, we apply the above steps for paths with more than a single reflection, e.g., ($R_{\max}\;⩾\;2$). The change is that additional mirroring occurs in step 1 and that the validity checks in step 2 are expanded for the path calculation.

**Figure 7:**
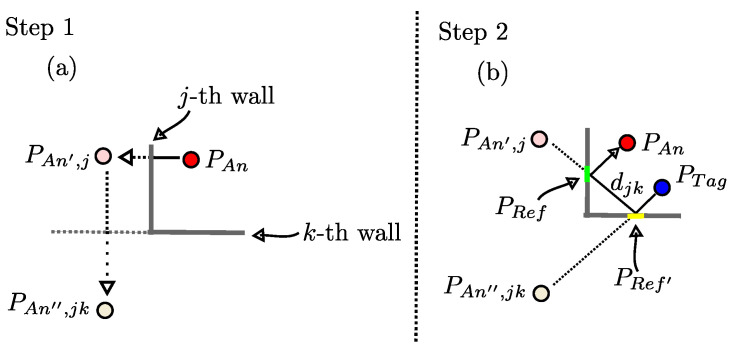
Expansion of the modeling for reflected signal paths with two reflections: (**a**) Step 1: Determination of additional virtual anchors. (**b**) Step 2: Calculation of the resulting reflected signal’s path.

In step 1, except mirroring the original anchor, we mirror the *j*-th virtual anchor at all remaining surfaces with k≠j, resulting in 2nd-order virtual anchor at $P_{{\mathrm{An}}^{\prime \prime },jk}$. Figure 7a shows how additional virtual anchors are created for additional reflections in the paths. In general, for $R_{\max}$ reflections on *J* surfaces, $J\cdot {\left(J-1\right)}^{R_{\max}}$ virtual anchors result.

So, overall two reflection points result. On the line $P_{{\mathrm{An}}^{\prime \prime },jk}$ to $P_{\mathrm{Tag}}$, $P_{{\mathrm{Ref}}^{\prime }}$ is its intersection with the *k*-th wall. $P_{\mathrm{Ref}}$ is the intersection of the line $P_{{\mathrm{An}}^{\prime }}$ to $P_{{\mathrm{Ref}}^{\prime }}$ with the *j*-th wall. The path length of the corresponding signal path is $d_{jk}=d_{\ell 2}\left(P_{\mathrm{Tag}}-P_{{\mathrm{An}}^{\prime \prime },jk}\right)$. Figure 7b depicts the path determination in step 2.

Finally, in step 3, this time three lines, namely $P_{\mathrm{An}}$ to $P_{\mathrm{Ref}}$, $P_{\mathrm{Ref}}$ to $P_{{\mathrm{Ref}}^{\prime }}$, and $P_{{\mathrm{Ref}}^{\prime }}$ to $P_{\mathrm{Tag}}$, are checked for interruption of the surface, as mentioned above.

Knowing all *I* valid paths and their respective lengths $\left\{d_{i}\right\}$, the needed characterization parameters $\hat{\tau_{i}}=d_{i}/c_{0}$ are determinable. The estimated delays are needed for modeling the receive signal. Also, they are included for the upcoming statistical amplitude analysis.

#### 3.3.2. Statistical Analysis of the Amplitude for Estimation

According to Equation (3), to realistically model the receive signal, we need the transmission delay ${\hat{\tau }}_{i}$ and amplitude ${\underline{a}}_{i}$ of each reflected signal. Compared to ${\hat{\tau }}_{i}$, the modeling of the complex amplitude ${\underline{a}}_{i}$ is more challenging [22], as it is influenced by external factors such as material- and angle-dependent reflection losses due to signal’s wall and obstacle penetration, and hardware-specific factors such as the automatic gain control (AGC) and the analog-to-digital converter (ADC) [27]. Also, the characteristics of the interference between two reflected signals is a further challenge which, under the given circumstances, makes it extremely difficult to trace back to specific amplitudes [29]. So, for SALOS, we will model the amplitude randomly. In the following, we analyze the distribution statistically and identify important factors of the result. Based on them, we describe the chosen randomized modeling of the amplitude.

**Note:** In the following, we call the direct path without reflecting the 0*th (reflection) order* path. The paths with 1 or 2 reflection points are called the 1*st and* 2*nd (reflection) order* paths.

**Analysis of amplitude’s characteristics and influence on modeling:** For the analysis of the amplitude’s characteristics, we recorded around 600 signal measurements for 20 tag and anchor position combinations, resulting in around 12,000 measurements. For these measurements, we determine the transmission delay of all reflected signals of the 0th, 1st, and 2nd reflection orders following Section 3.3.1.

**Figure 8:**
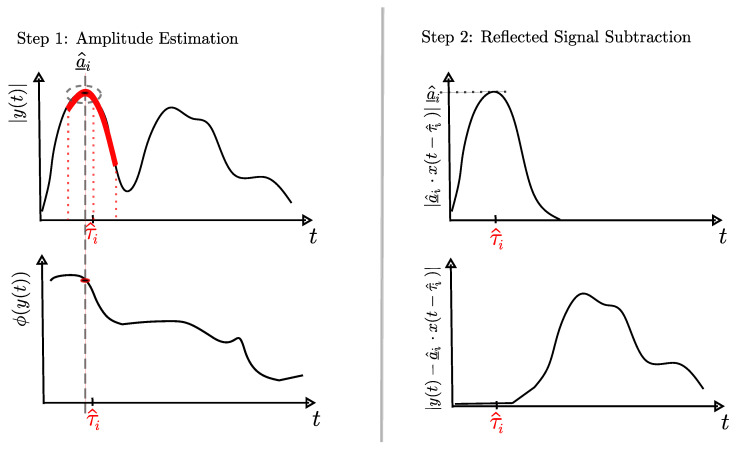
Process for iterative complex amplitude estimation ${\underline{\hat{a}}}_{i}$ of *i*-th reflected signal.

We estimate the complex amplitude ${\underline{a}}_{i}$ of the reflected signals iteratively in magnitude $|{\underline{a}}_{i}|$ and phase $\phi ({\underline{a}}_{i})$. To analyze the amplitude with decreasing receive power, we consider the 0th order reflected signal first, then the reflections of the 1st order with increasing transmission delay, and finally, the reflected signals of the 2nd order also with increasing transmission delay. The following steps are identical for all reflected signals, as shown in Figure 8. First, we determine the analysis interval $\left[\hat{\tau_{i}}-1\;\mathrm{ns},\hat{\tau_{i}}+1\;\mathrm{ns}\right]$, to compensate for discrepancies between the measurement setup and modeling of up to 30 cm (=1 ns). Within this interval, we find the highest magnitude of the measured signal. For this maximum, we save the amplitude in magnitude and phase as ${\hat{\underline{a}}}_{i}$. If there was no maximum in the interval, but only a continuous curve, we save the complex amplitude at $\hat{\tau_{i}}$. Finally, we subtract the shaped reflected signal ${\hat{\underline{a}}}_{i}\cdot x\left(t-\hat{\tau_{i}}\right)$ from the measured signal and process this difference further with the next reflected signal.

Note: The following analysis is intended to provide an overview of the amplitudes for the given model. For a more realistic characterization of the amplitude, the distribution must be examined more closely. But here, indicators are enough for us to accurately model the complex amplitude successfully for the artificial receive signals.

We classify the amplitudes according to 0th (around 12,000 values), 1st (around 60,000 values), and 2nd order (around 120,000 values) reflections. For analysis, we divide the complex values into phase and magnitude.

With few deviations, the phase estimations of the 0th, 1st, and 2nd order reflections are located in the interval [0,2π]. There is no correlation between transmission delay and phase value; therefore, these phases are considered independent of the transmission delay.

**Figure 9:**
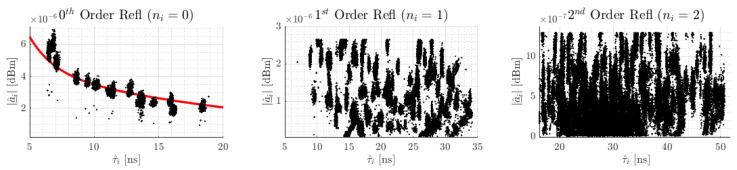
Results of the magnitude’s estimation $|{\hat{\underline{a}}}_{i}|$ of the 0th, 1st, and 2nd order reflected signals.

The results of the magnitude estimation are shown in Figure 9. From left to right, it depicts the magnitude estimations for all 0th, 1st, and 2nd order reflected signals with respect to the estimated transmission delay ${\hat{\tau }}_{i}$. As expected following Friis’ path loss model from [30], the direct path shows an exponential decay of the magnitude with increasing distance between the sensor nodes. The red line in the plot depicts the exponential fitting with respect to the path losses. Table 1 lists the resulting fitting. The magnitudes of the 1st and 2nd order reflection are independent of the transmission delay. For them, the distribution of the magnitude is randomly between 0 and a maximum value: 1st order maximum at $2.7\times 10^{-6}$ dBm, 2nd order maximum at $1.3\times 10^{-6}$ dBm. We do not know the reason for this random distribution, but we take the corresponding maximum values as characteristics for modeling in our approach.

Based on the given characteristics, we determine random numbers for the magnitude and phase of each reflected signal. Each phase is assumed to be uniformly distributed with $\phi \left(\hat{{\underline{a}}_{i}}\right)\in U\left(\left[0,2\pi \right]\right)$. In general, for magnitude modeling, we assume the 0th order reflected signals to match the fitting described above. The magnitudes of 1st and 2nd order are normally distributed with $|\hat{{\underline{a}}_{i}}|\in N\left(\mu ,\sigma \right)$. For this, we determine mean μ and standard deviation σ based on the given sets of magnitudes. For these magnitudes, we also limit the random numbers upwards and downwards. If one of the random numbers is outside this range, the number is rolled again until the magnitude is within the range. The description of the random modeling for the 0th, 1st, and 2nd order reflections is listed in Table 1:

At this point, models for the transmission delays and the complex-valued amplitude are presented. Now the construction of the modeled receive signals and the allocation to signal sets for reference follows.

#### 3.3.3. Modeling of the Receive Signals Sets for Reference

As mentioned above, to accurately model artificial receive signals, we need the UWB transmit signal x(t) as well as a realistic CIR. With the given estimates for ${\hat{\underline{a}}}_{i}$ and $\hat{\tau_{i}}$, we construct the CIR $\hat{h}\left(t\right)$. Following Equation (3), with respect to the CP of the tag, $\hat{h}\left(t\right)$ becomes $h_{\mathrm{CP}}\left(t\right)$ and the modeled signal $y_{\mathrm{CP}}\left(t\right)$ is calculated by convolution of x(t) and $h_{\mathrm{CP}}\left(t\right)$:

(6)yCP(t)=x(t)∗hCP(t)(7)=∑i=0I−1|a_^i|·ej·ϕ(a_^i)︸a_^i·x(t−τi^)

Including three-dimensional multipath modeling as shown before, $y_{\mathrm{CP}}\left(t\right)$ provides a realistic estimate for the omnidirectional received signal between the anchor at $P_{\mathrm{An}}$ and the tag at the corresponding CP.

**Figure 10:**
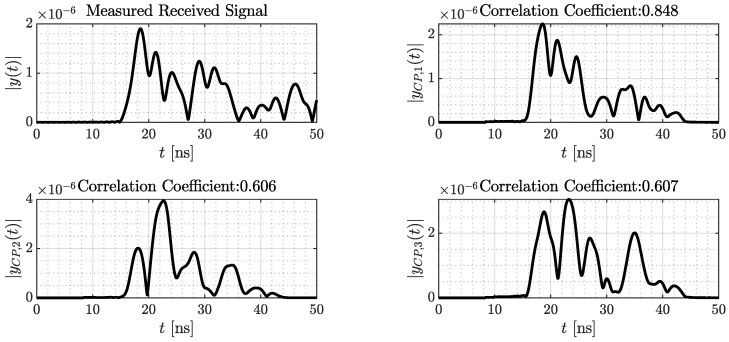
Shape of measured signal compared to three different modeled signals of the same tag position.

The random amplitudes are crucial for the final shape of the modeled signals. Figure 10 shows at the top left a measured signal for a known spatial geometry, tag, and anchor position. The other receive signals shown are modeled based on the same setup with varying estimations of the complex amplitudes ${\hat{\underline{a}}}_{i}$. The respective correlation coefficient is given in the plot titles. While the shape at the top right seems to fit well, the modeled signals in the bottom row differ significantly from the measured one. This results only from the mis-modeled CIRs.

A good variance within the modeling increases the probability of correctly estimating the corresponding measured signal. For this, we create sets of $N_{\mathrm{CP}}^{y(t)}$ different randomly modeled signals per CP. These sets serve as reference data for SALOS.

After the description of artificial reference data modeling, the next section depicts how the localization with majority decision is performed.

## 4. Localization Algorithm

The implementation of the localization algorithm in relation to the underlying input data and hardware is decisive for the accuracy of the system. Based on the modeled receive signal set, we design the localization algorithm and its implementation with Qorvo’s DW1000 UWB RF chip, integrated for measurement. In the first step, we derive the optimal anchor position for a given spatial geometry in Section 4.1. The DW1000 provides a raw UWB receive signal, which needs some processing, as shown in Section 4.2. Finally we depict the localization algorithm based on the comparison of a measured receive signal and the modeled signal set in Section 4.3.

### 4.1. Optimal Position of the Anchor

In Section 3.2, we define it such that all tag positions must result in unambiguous CIRs with respect to our chosen anchor position to avoid the need of additional assumptions, measured information, or sensors. To place the anchor with respect to this case, in our previous work [13], we introduced the *effective length of CIRs* $\ell_{e}$ as a measure of the analyzed time interval to achieve unambiguity of CIRs.

In general, two conditions for an anchor position are derived for optimization:

- A valid anchor position results in unambiguous CIRs for all tag positions.
- The optimal anchor position achieves the unambiguity of all CIRs with the shortest effective length $\left[0,\ell_{e}\right]$.

We always consider the optimal anchor position with respect to the real tag positions as well as to all candidate points to ensure unambiguity.

### 4.2. Signal Processing of Qorvo’s DW1000 Raw Measurements

The UWB receive signal measurement serves as input for the localization algorithm. This is realized by transmission via Qorvo’s DW1000 RF chip. But the DW1000 does not provide the measurement as needed, e.g., without distance information and accurate amplitude; therefore, we process the signal after recording.

We apply a two-way ranging (TWR) for estimating the distance $d_{0}$ between anchor and tag, which is described in Appendix A. $d_{0}$ correlates to the transmission delay of the direct path with $\tau_{0}=d_{0}/c_{0}$, and $c_{0}\approx 3\times 10^{8}$ is speed of light. Additionally, we read the receive signal from the final message of TWR. In the following, $y_{\mathrm{raw}}\left[mT_{s,\mathrm{raw}}\right]$ is this DW1000’s measured signal with *M* samples, m=[0,...,M−1]. The sample time is $T_{s,\mathrm{raw}}\approx \;1$ ns. Since $y_{raw}\left[mT_{s,\mathrm{raw}}\right]$ is a time-shifted, normalized, discrete, baseband-version of y(t), we need further signal processing to recover y(t). We describe the signal processing in this section.

The DW1000 marks the beginning of the first reflected signal with the help of a leading-edge algorithm. The resolution of this marker is in the range of 1/64 ns, achieved by oversampling. With the transmission delay $\tau_{0}$, we are able to create a matching time axis for the measurement.

Also, the DW1000 stores the measured receive signal strength (RSS) of the signal $P_{\mathrm{Rx}}$ in dBm. With $P_{\mathrm{Rx}}$, we reconstruct the original output level of y(t).

Finally, we interpolate the signal to estimate the data gaps between the samples. For interpolation, we convolve it with a sinc-function with zero crossings spaced at $T_{s,\mathrm{raw}}$. Therefore, our processed signal is y(t):

(8) $$\begin{array}{c} y\left(t\right)=\underset{\mathrm{Output}\;\mathrm{Level}\;\mathrm{Reconstruction}}{\underset{︸}{\frac{10^{\left(\frac{P_{\mathrm{Rx}}}{10}\right)}}{\sum_{m=0}^{M-1}{\left|y_{\mathrm{raw}}\left[mT_{s,\mathrm{raw}}\right]\right|}^{2}}}}\cdot \underset{\mathrm{Interpolation}}{\underset{︸}{\sum_{m=0}^{M-1}y_{\mathrm{raw}}\left[mT_{s,\mathrm{raw}}\right]*\frac{\sin(\pi t/T_{s,\mathrm{raw}})}{\pi t/T_{s,\mathrm{raw}}}}}. \end{array}$$

**Figure 11:**
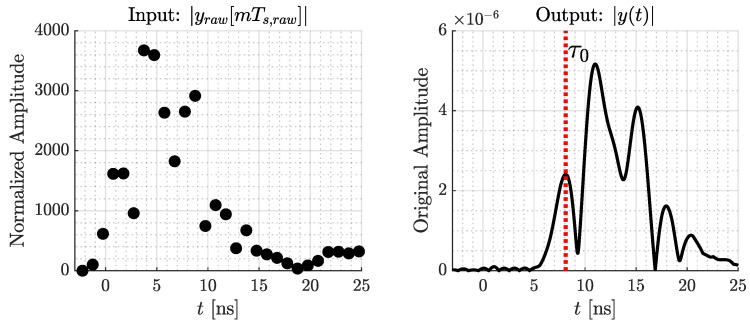
Signal processing steps for DW1000’s measurement $y_{\mathrm{raw}}\left[mT_{s,\mathrm{raw}}\right]$ to the reconstructed y(t).

Figure 11 depicts the input and output of our signal processing.

For real implementation, we realize the interpolation with a discrete sinc-function in the range of −10 ns to 10 ns with a sample time of $T_{S}=1/64$ ns. Following Equation (8), this results in $y[kT_{S}]$ with *K* samples indicated by k=0,...,K−1. In the following, this processed measurement is called the measured signal.

After modeled and measured signal are prepared, we focus on the position estimation based on the similarity between the measured and modeled signals.

### 4.3. Majority-Based Position Estimation

In SALOS, we compare the measured signal y(t) with sets of modeled signals $\left\{y_{\mathrm{CP}}\left(t\right)\right\}$ to find the most likely CP for position estimation. For determining this estimate for the given signals, a proper similarity metric focusing on the shape of the signals is needed. Otherwise, the reflected signal positions and therefore the multipath propagation lose importance.

The position estimation of the localization is divided into two steps. First, the most similar modeled receive signal for the measured signal is determined. It indicates the most probable tag position. This is performed for multiple sets of modeled signals. In the second step, the position estimations of the distinct signal sets are evaluated via majority decision to finally estimate the tag’s position.

For signal comparison, we implement the discrete cross-correlation as a similarity metric. **Note:** Each modeled signal $\left\{y_{\mathrm{CP}}\left(t\right)\right\}$ is also sampled with $T_{S}\;\approx \;1/64$ ns and consists of *K* samples with k=0,...,K−1. The time axis of the measured and modeled signal is identical.

$d_{S}\left[n\right]$ is the correlation coefficient between $y[kT_{S}]$ and $y_{\mathrm{CP},n}\left[kT_{S}\right]$ of the *n*-th CP with:

(9) $$\begin{array}{c} d_{S}\left[n\right]=\frac{1}{K-1}\sum_{k=0}^{K-1}\left(\frac{y\left[kT_{s}\right]-\mu \left(y\left[kT_{s}\right]\right)}{\sigma (y\left[kT_{s}\right])}\right)\left(\frac{y_{\mathrm{CP},n}\left[kT_{s}\right]-\mu \left(y_{\mathrm{CP},n}\left[kT_{s}\right]\right)}{\sigma (y_{\mathrm{CP},n}\left[kT_{s}\right])}\right), \end{array}$$

where μ(·) is the vector’s mean value and σ(·) its variance. The more similar the signals, the higher the correlation coefficient.

We estimate the position using the nearest neighbor concept; the best position estimate $\hat{P}$ among the CPs also has the most similar modeled signal:

(10) $$\begin{array}{c} \hat{P}\;=\;\underset{n\;=\;0,\;...\;,\;N-1}{arg max}d_{S}. \end{array}$$

**Figure 12:**
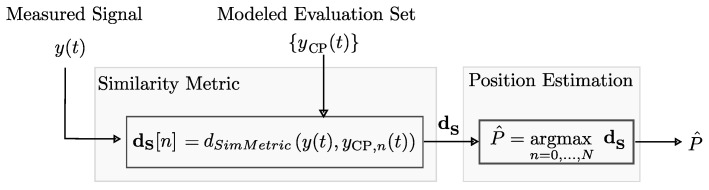
General structure of position estimation for one evaluation set including $N_{\mathrm{CP}}^{y(t)}\times N_{\mathrm{CP}}$ modeled signals.

As mentioned in Section 3.3, $N_{\mathrm{CP}}^{y(t)}$ signals are modeled randomly for each CP. If $N_{\mathrm{CP}}$ is the number of CPs, a complete modeled evaluation set includes $N_{\mathrm{CP}}^{y(t)}\times N_{\mathrm{CP}}$ modeled signals. The overall structure of SALOS’s position estimation for one evaluation set is shown in Figure 12.

Increasing $N_{\mathrm{CP}}^{y(t)}$ leads to advantages and disadvantages of the comparison. On the one hand, there is a higher probability of modeling a signal with a realistic shape for a specific CP. But in general, increasing $N_{\mathrm{CP}}^{y(t)}$ also increases the probability for an unfavorable mis-modeling of a false CP’s signal. So, although the mapping from signal to tag position is bijective (see Section 3.2), the random-based signal modeling leads to an ambiguous signal under an unfortunate seed. Larger sets are therefore not directly associated with an increasing number of correct position estimates.

To lower this influence on the position estimation, multiple independent modeled evaluation sets are created. For each of the sets, the tag position ${\hat{P}}_{l}$ is estimated as described above. The overall position estimation ${\hat{P}}_{\mathrm{MD}}$ is performed by majority decision of these estimations:

- If a majority of the estimates are identical, then this is also the position estimation ${\hat{P}}_{\mathrm{MD}}$.
- If several positions are estimated equally often, then the correlation coefficients of the corresponding estimates are compared. The highest coefficient indicates ${\hat{P}}_{\mathrm{MD}}$.

After position estimation, the localization starts with the next measurement without changing the evaluation sets. So, only the DW1000 signal processing and the comparison itself need to be repeated per measurement. Note that we also implemented the described structure of SALOS modularly with real hardware. Appendix B describes the implementation details. We also provide the datasets of the modeled and measured signals for download: https://git.mylab.th-luebeck.de/sven.ole.schmidt/salos-2024 (accessed on 3 April 2024).

The evaluation of position estimation accuracy follows in the next section.

## 5. System Evaluation

Previously, we showed that position estimation is possible with SALOS in general. In this section, we will analyze to what extent the integration of a completely model-based reference influences the position estimation accuracy. Without proper accuracy, SALOS is not an alternative to established systems, despite hardware minimization. A valid evaluation of the position estimation accuracy is dependent on a realistic setup and a comparison benchmark, as described in Section 5.1. The metric for evaluation and the resulting accuracy are shown in Section 5.2 and discussed in Section 5.3.

### 5.1. Evaluation Setup

The design of the evaluation setup significantly impacts the validity of position estimation accuracy results. Next, we will describe the implementation for evaluation, covering the hardware, the environment, and the comparison setups.

**Figure 13:**
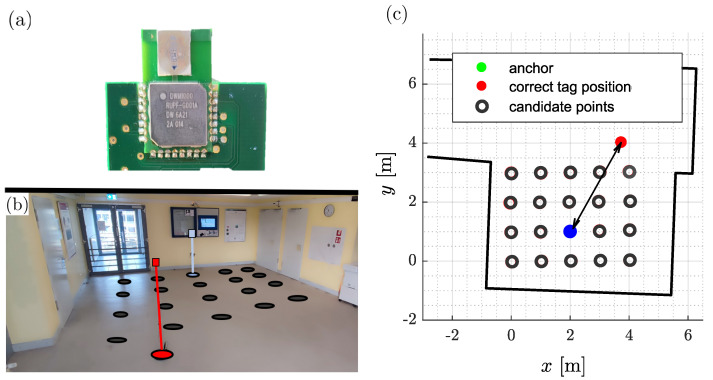
Evaluation setup for SALOS: (**a**) Qorvo’s DW1000 RF Chip, equipped on sensor node. (**b**) Real indoor office corridor. (**c**) Evaluation set of CPs: original tag positions.

Sensor nodes equipped with Qorvo’s DW1000 RF chip serve as tags and anchors (see Figure 13a). They are configured with the settings in Table 2. As described in Section 3.1, we will communicate on UWB channel 1 with a center frequency $f_{c}$ of 3.4944 GHz and a bandwidth *B* of 499.2 MHz. We already depicted the corresponding transmit signal in Figure 2. The additional settings for pulse repetition and data rate as well as preamble settings are listed in Table 2 for completeness.

These devices will be deployed on a floor in a building for evaluation. A large corridor is an excellent choice for this purpose, since there is a large area for node placement, which is characterized by various reflector materials and a non-rectangular spatial geometry. Note that we have measured and modeled the geometry in three dimensions. Figure 13b depicts the corridor.

Figure 13c depicts the footprint of the area, including the anchor position and the 20 selected tag positions. Additionally, the anchor position and the 20 tag positions are listed in Table 3. The tag positions cover an area of 3m×4m. This grid results from previously recorded measurements of other localization systems in our research group. The selected tag positions are therefore well-known, even though they are distorted in the room geometry. However, this does not affect the results. The anchor has been optimally placed with respect to these 20 positions (see Section 4.1).

Overall, we measure 500 receive signals at the 20 tag positions each. Since the anchor results in an effective length of $\ell_{e}\approx 40$ ns, we compare the first 40 ns of both the measured and modeled signals. For each measured signal, a position is estimated, resulting in 10,000 estimations to evaluate.

The modeled CPs are identical with the chosen tag positions. Figure 13c also illustrates this set. The 20 CPs are adequate for assessing the position estimation capabilities of the proposed localization system. By optimally placing the anchor in relation to these positions, distinguishing among the 20 positions should be feasible. It is possible to use a significantly higher number of CPs to determine the position if the anchor has been optimally placed in relation to the CPs.

For each CP, the reflected signal paths are modeled with $n_{i}⩽R_{\max}⩽2$ reflections per path. A higher number of paths increases processing complexity, e.g., for a six-sided spatial geometry, 6+30 paths with a maximum of 2 reflections result, while there are an additional 150 paths with exactly 3 reflections. Considering the reflected signals passing through those paths, further tests showed us that the receive signal strength of these signals is too low compared to paths with $R_{\max}\;⩽\;2$. This results from additional reflection losses and in general longer reflection paths. Including these reflection signals in the modeling acts as an additional source of noise that degrades the system. So, we only include paths with $R_{\max}\;⩽\;2$ reflections, to reduce the complexity for calculation and therefore processing time.

Choosing this set of CPs results in the optimal setup to evaluate the position estimation accuracy of the localization system. We construct four evaluation sets, including 50 modeled signals per CP. This results in $N_{\mathrm{CP}}^{y(t)}\times N_{\mathrm{CP}}=50\times 20=1000$ modeled signals per set. The modeled receive signals are calculated in advance and then made available to the localization system. E.g., for our setup, the calculation time of the individual modeled receive signals is in the range of approx. 60 ms. Due to parallel computing, a set of 50 modeled signals can therefore be created in approx. 600 ms. Note: Applying this evaluation setup, both the measured and modeled receive signals result from three-dimensional multipath propagation.

As part of the evaluation, we aim to determine if integrating multipath propagation into the modeled receive signals alone supports position estimation. Therefore, we compare it with two settings that lack this integration. So, we compare the estimation accuracy with two randomized estimations as a benchmark for evaluation. For the first, we calculate the distance between each tag position and all CPs (see Figure 13c). The histogram of the resulting distances represents guessing the CPs without any information, e.g., randomly. This is the scenario when none of the modeled multipaths are supporting. The second setting covers randomized position estimations for CPs with a similar distance to the anchor as the correct estimate. This scenario is when at least the distance estimation is correct, but none of the modeled multipath information is useful. These randomized estimations set the lower threshold of accuracy to indicate the amount of information modeled.

### 5.2. Evaluation of the Position Estimation Accuracy

For evaluation of the estimation accuracy, we calculate the estimation error based on the Euclidean distance $d_{\ell 2}\left(P_{\mathrm{Tag}},{\hat{P}}_{\mathrm{Tag}}\right)$ between the actual tag position $P_{\mathrm{Tag}}=\left[x,y,z\right]$ and the estimated position ${\hat{P}}_{\mathrm{Tag}}=\left[\hat{x},\hat{y},\hat{z}\right]$ for each measured signal:

(11) $$\begin{array}{c} d_{\ell 2}\left(P_{\mathrm{Tag}},{\hat{P}}_{\mathrm{Tag}}\right)=\sqrt{{\left(x-\hat{x}\right)}^{2}+{\left(y-\hat{y}\right)}^{2}+{\left(z-\hat{z}\right)}^{2}}. \end{array}$$

**Figure 14:**
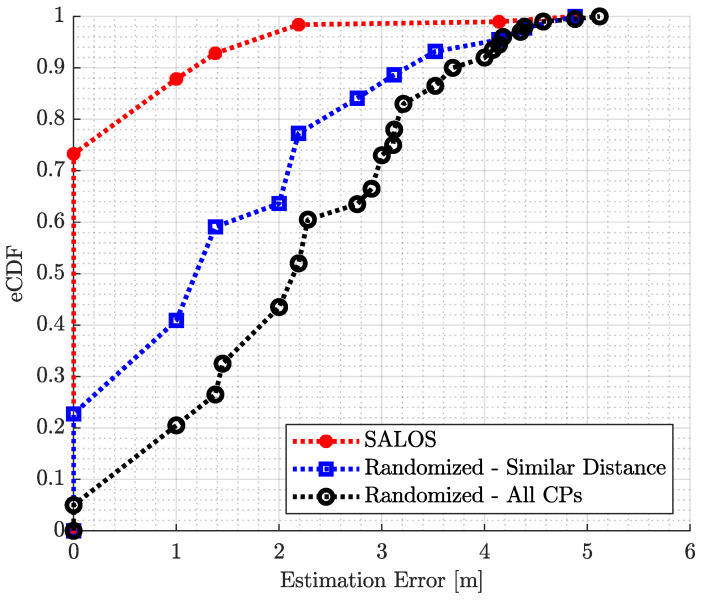
eCDF of position estimation errors for SALOS, as well as for a randomized positioning pick with and without including distance estimation.

The resulting position estimation errors are presented as empirical cumulative distribution functions (eCDFs). The eCDFs of estimation errors and of the randomized estimation errors are shown in Figure 14.

The black eCDF depicts the position errors for the set, including any CP. For the blue eCDF, the distance estimate is integrated as a preselection of CP. While the overall random set makes a total correct decision of 5% for all 20 CPs, the inclusion of the distance estimate in the random selection increases the number of correct decisions to around 23%. Both graphs then increase linearly. The completely random selection achieves an estimation error of a maximum of 1 m in 21% and an error of a maximum of 2.2 m in 52%. With a reduced number of CPs, the position estimation achieves an accuracy of 1 m in 41% of the cases and 2.2 m in 77% of the position estimates. The maximum estimation error is 5.12 m for the black eCDF and 4.88 m for the blue eCDF.

The red eCDF shows the result of the SALOS position estimation errors. A total of 7327 measurements and thus around 73% of the positions are correctly estimated by incorporating modeled multipath propagation. Almost 88% of the estimates have a maximum deviation of 1 m. More than 9840 of the 10,000 measurements were traced back to the correct position with an accuracy of 2.2 m. The maximum estimation error is 4.88 m, like the blue eCDF.

### 5.3. Discussion

The motivation behind SALOS is to improve a single-anchor localization system through sophisticated signal modeling. With modeled receive signals as reference measurements, we achieve position estimation without further external measured information.

With a pure random estimation, we achieve 5% (one out of twenty) correct position estimations. With the help of the line-of-sight signal distance estimation, the accuracy is improved to 23%. With our approach to multipath signal modeling, the accuracy increases to 73%. So, the number of correct position estimates is improved by a factor of 3.17 and 14.5, respectively, which demonstrates the significant advancement of the modeling.

There are certain positions with high estimation errors. These are positions that have a strong first-order reflection in the area of the glass door shown in Figure 13c. Although we do not consider wall material in our model, this effect is a strong indicator to consider glass walls in the 3D model.

After discussion of the evaluation results, we conclude this work with a summary and a view on future tasks regarding SALOS.

## 6. Conclusions and Future Work

In this paper, we presented SALOS, a modular model-based UWB system for indoor single-anchor localization. SALOS is a novel approach that reduces the infrastructure and costs of conventional multi-anchor systems by exploiting the multipath propagation of the UWB signal. We developed new algorithms for SALOS, such as the three-dimensional multipath model, creating artificial receive signal models as reference for localization, and the majority decision-based position estimation. We evaluated SALOS on a floor in a building with 20 distinct tag positions and achieved correct position estimations for more than 73% of the measurements. Our results demonstrate the feasibility and accuracy of SALOS for indoor localization. In particular, the influence of the realistic modeled receive signals is shown in the results. However, SALOS also faces some challenges, such as the placement of the anchor, the sensitivity to environmental changes, and the scalability to larger areas.

Therefore, we suggest some possible improvements in future work for SALOS. To maximize the multipath diversity and coverage, we will optimize the anchor placement, which includes both position and orientation. Also, we will adapt the multipath model and the artificial signal models to accommodate dynamic and heterogeneous environments, including tag positions with non-line-of-sight conditions. The position determination follows a very simple approach that ignores the relative similarity based on the correlation coefficient. The influence of weighting the candidate points based on similarity will be examined in the future. In the future, we plan to explore techniques, such as machine learning, deep learning, and cooperative localization, to see if and how much they could enhance the system’s capabilities and performance. In addition, it is of interest to find out how accurately SALOS can be implemented as a multi-cell system using several optimally placed anchors. Also, for future live implementation, our approach needs optimization for real-time operation with high update rates. We will conduct experiments with multiple tags to scale up the system and improve reliability and redundancy. We need to evaluate the system performance in different indoor settings, including a higher number of tag positions that are closer together. We will compare it with other state-of-the-art systems, like UWB multi-anchor systems and Bluetooth AoA systems.

The concept of SALOS can be extended to work with multiple antennas, as depicted in the related work section. We hope that our work will inspire more research and innovation in the field of UWB single-anchor indoor localization.   

## Figures and Tables

**Table 1:** Distribution characterization of the amplitudes in magnitude and phase with respect to the reflection order.

Order of Reflection	Magnitude $|{\hat{\underline{a}}}_{i}|$	Phase $\phi ({\hat{\underline{a}}}_{i})$
0th	$3.04\times 10^{-5}\cdot e^{-5.22\times 10^{8}\cdot \tau_{i}}+5.38\times 10^{-6}\cdot e^{-4.81\times 10^{7}\cdot \tau_{i}}$	U([0,2π])
1st	$0⩽N\left(1.24\times 10^{-6},7.46\times 10^{-7}\right)⩽2.66\times 10^{-6}$	U([0,2π])
2nd	$0⩽N\left(4.12\times 10^{-7},3.30\times 10^{-7}\right)⩽1.30\times 10^{-6}$	U([0,2π])

**Table 2:** Selected settings of Qorvo’s DW1000 RF chip.

Setting	Value
UWB channel	1
Center frequency $f_{c}$	3.4944 GHz
Bandwidth *B*	499.2 MHz
Pulse repetition frequency	64
Preamble length	128
Preamble acquisition chunk size	8
Preamble code anchor and tag	9
Data rate	6.8 MBit/s

**Table 3:** Real tag and anchor positions.

Name	Coordinates [x,y,z]	Name	Coordinates [x,y,z]
$P_{\mathrm{Tag},11}$	[0.0,0.0,1.37]	$P_{\mathrm{Tag},31}$	[0.0,2.0,1.37]
$P_{\mathrm{Tag},12}$	[1.0,0.0,1.37]	$P_{\mathrm{Tag},32}$	[1.0,2.0,1.37]
$P_{\mathrm{Tag},13}$	[2.0,0.0,1.37]	$P_{\mathrm{Tag},33}$	[2.0,2.0,1.37]
$P_{\mathrm{Tag},14}$	[3.0,0.0,1.37]	$P_{\mathrm{Tag},34}$	[3.0,2.0,1.37]
$P_{\mathrm{Tag},15}$	[4.0,0.0,1.37]	$P_{\mathrm{Tag},35}$	[4.0,2.0,1.37]
$P_{\mathrm{Tag},21}$	[0.0,1.0,1.37]	$P_{\mathrm{Tag},41}$	[0.0,3.0,1.37]
$P_{\mathrm{Tag},22}$	[1.0,1.0,1.37]	$P_{\mathrm{Tag},42}$	[1.0,3.0,1.37]
$P_{\mathrm{Tag},23}$	[2.0,1.0,1.37]	$P_{\mathrm{Tag},43}$	[2.0,3.0,1.37]
$P_{\mathrm{Tag},24}$	[3.0,1.0,1.37]	$P_{\mathrm{Tag},44}$	[3.0,3.0,1.37]
$P_{\mathrm{Tag},25}$	[4.0,1.0,1.37]	$P_{\mathrm{Tag},45}$	[4.0,3.0,1.37]
$P_{\mathrm{An}}$	[3.6,4.17,1.37]		

**Figure A1:**
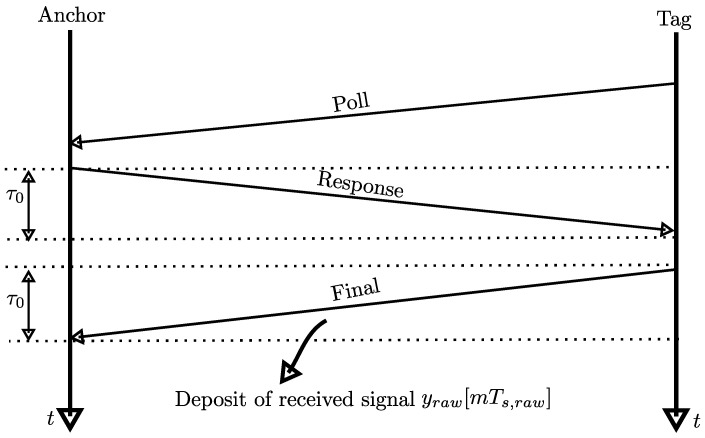
Two-way ranging for distance estimation based on the DW1000 RF chip.

**Figure A2:**
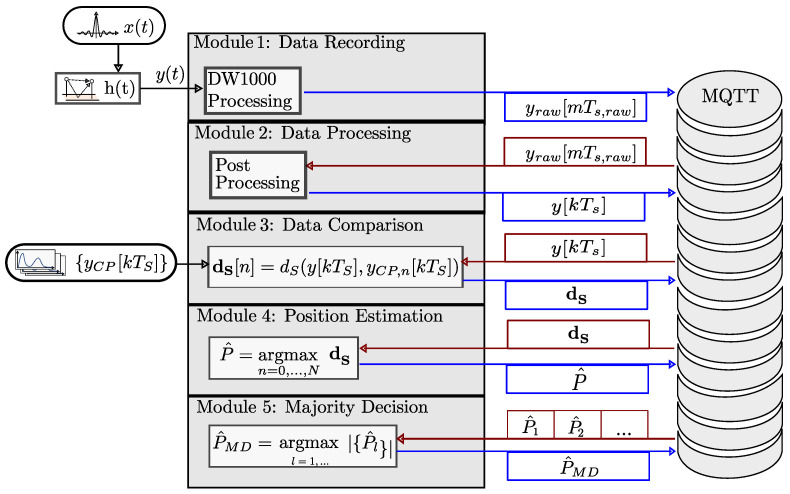
Block diagram of the MQTT-based modular live implementation.

## Data Availability

We provide the datasets of the modeled and measured signals for reconstructing the results of Section 5 in the following repository: https://git.mylab.th-luebeck.de/sven.ole.schmidt/salos-2024 accessed on 3 April 2024.

## Abbreviations

    The following abbreviations are used in this manuscript:

ADC	analog-to-digital converter
AGC	automatic gain control
AoA	angle-of-arrival
CIR	channel impulse response
COTS	commercially off-the-shelf
CP	candidate point
eCDF	empirical cumulative distribution function
ESPAR	electronically steerable parasitic array radiator
IMU	inertial measurement unit
MQTT	message queuing telemetry transport protocol
PDoA	phase difference of arrival
RF	radio frequency
RSS	receive signal strength
SALOS	single anchor localization system
TDoA	time difference of arrival
TWR	two-way-ranging
UWB	ultra-wideband
3D	three-dimensional

## Appendix A. DW1000 Distance Estimation via Two-Way Ranging

For distance estimation, we implemented the two-way ranging method provided by the DW1000 described in [31]. Figure A1 sketches the message exchange necessary for this. After an initial synchronization, the anchor receives a Poll message from the tag to start the TWR. The anchor responds with a Response message, to which the tag responds with a Final message. The time interval between sending the Response and receiving the Final message is twice the Time of Flight $\tau_{0}$ including the duration of the tag’s processing, which is known to the anchor.

## Appendix B. The Modular Live-Streaming Structure of SALOS

The setup shown in the previous sections allows SALOS to be implemented using the DW1000 RF chip. The system can be operated live through the commissioning and the continuous data recording. To achieve a sufficiently high update interval for the position estimate, it is advisable to carry out the processing steps in parallel. In addition, the scalability of the system must be guaranteed, which is required by a large number of CPs and several tag sensor nodes. Since clearly separate processing steps result, the processing is divided into modules.

Figure A2 shows the selected structure. SALOS was divided into four modules that communicate with each other via the message queuing telemetry transport protocol (MQTT). MQTT is a lightweight communication protocol specifically designed to efficiently transfer messages between devices with limited resources. At the center is an MQTT broker that serves as a data hub. The modules take on at least one of two roles. The publisher stores data with a topic (e.g., SALOS/DW1000Data/Tag1/) on the data hub. The format of the data is selected flexibly, since the data are not interpreted on the broker. We have decided on a binary format for the data, because in contrast to ASCII characters, the transmission speed is increased [32]. The subscriber is able to subscribe to topics freely. Including a wildcard character # subscribes to all subtopics of one topic (e.g., SALOS/DW1000Data/# orders a subscription to all subtopics in the main topic SALOS/DW1000Data/). When data are stored on a subscribed topic, the data will be sent to the subscriber immediately when it arrives. The modules take on one or even both roles at the same time, which makes it easy to implement continuous data processing. With this communication protocol, new modules or external viewers are integrated without great effort. Note that the broker has no memory, so only the latest message of a topic is available for retrieval.

So, the modules of SALOS are exchangeable and scalable via live usage without intersecting the overall system. The data recording by the sensor node with built-in DW1000 RF chip is placed in module 1, described in Section 4.2. In module 2, the receive signals are post-processed (cf. also Section 4.2). The application of the chosen similarity metric (see Section 4.3), including the stored receive signals of the CPs (as generated in Section 3.3.1), is in module 3. In the fourth and last module, the position of the tag is estimated using the similarity metric’s results (also covered in Section 4.3).
